# Fluid mechanics approach to analyzing collagen fiber organization

**DOI:** 10.1117/1.JBO.27.1.016503

**Published:** 2022-01-31

**Authors:** Adriana C. Salazar Coariti, Maurice S. Fabien, Johnny Guzman, Jeffrey A. McGuire, Raffaella De Vita, Kimani C. Toussaint

**Affiliations:** a Brown University, PROBE Lab, School of Engineering, Providence, Rhode Island, United States; b Brown University, Division of Applied Mathematics, Providence, Rhode Island, United States; c Virginia Tech, Soft Tissue Research: Experiments, Theory, and Computations by Hokies (STRETCH) Lab, Department of Biomedical Engineering and Mechanics, Blacksburg, Virginia, United States

**Keywords:** vector fields, collagen, second-harmonic generation, waviness, fluid mechanics

## Abstract

**Significance:**

The spatial organization of collagen fibers has been used as a biomarker for assessing injury and disease progression. However, quantifying this organization for complex structures is challenging.

**Aim:**

To quantify and classify complex collagen fiber organizations.

**Approach:**

Using quantitative second-harmonic generation (SHG) microscopy, we show that collagen-fiber orientation can be viewed as pseudovector fields. Subsequently, we analyze them using fluid mechanic metrics, such as energy U, enstrophy E, and tortuosity τ.

**Results:**

We show that metrics used in fluid mechanics for analyzing fluid flow can be adapted to analyze complex collagen fiber organization. As examples, we consider SHG images of collagenous tissue for straight, wavy, and circular fiber structures.

**Conclusions:**

The results of this study show the utility of the chosen metrics to distinguish diverse and complex collagen organizations. We find that the distribution of values for E and U increases with collagen fiber disorganization, where they divide between low and high values corresponding to uniformly aligned fibers and disorganized collagen fibers, respectively. We also confirm that the values of τ cluster around 1 when the fibers are straight, and the range increases up to 1.5 when wavier fibers are present.

## 1 Introduction

Collagen is the primary structural protein in the extracellular matrix and in connective tissue.^1^^,^^2^ The organization of collagenous fibers could be used as a biomarker for structural anomalies, disease diagnosis and progression, aging, tissue development, and damage.^1^^,^^3^^–^^7^ The noncentrosymmetric molecular assembly of fibrillar collagen produces a nonlinear optical response when it interacts with light where two input photons produce an output photon with twice the frequency, a process known as second-harmonic generation (SHG).^8^ Therefore, high contrast images of collagenous fibers without exogenous staining can be obtained through SHG microscopy. Various types of quantitative SHG imaging methods have been used to contribute to understanding the role of collagen microstructure in biological function.^4^^,^^6^^,^^9^^–^^11^ For example, the texture analysis methods have been used to quantitatively classify SHG images for normal and cancerous human pancreatic tissues.^12^^,^^13^ Transforms, such as curvelet, wavelet, and Fourier, have also been used to quantify morphological features of collagen fibers.^4^^,^^14^^,^^15^ In the latter case, Fourier transform-second harmonic generation (FT-SHG) imaging analysis has been used to determine the orientation and spatial dispersion of uniformly organized collagen fibers, as in the case of tendon tissues.^16^ For disorganized collagen fibers, a grid-based approach is often used such that Fourier-transform analysis is carried out in local homogeneous regions, and the relative contributions of all regions are captured in a histogram of orientations.^3^^,^^4^^,^^6^^,^^16^^–^^18^ In two-dimensions (2D), the circular variance (CV) and the standard deviation (SD) of fiber orientation distribution have been used to distinguish highly aligned fibers from wavy fibers.^17^^,^^19^ Although FT-SHG provides insight on the types of morphology within an image, rapid changes in local features are not captured by the CV or SD alone.

Further understanding of spatially complex fiber organization can be found by reformulating this problem in the framework of a vector field (VF) map and leveraging the tools of computational fluid dynamics. VF analysis has been applied to understand diverse problems such as human mobility, fiber reconstruction, and bacteria collective motion. In one study, recurrent human mobility flow has been captured and simulated through a mesoscopic VF, which could facilitate the understanding of the spread of an epidemic in a region as well as help with urban planning.^20^ In another study, nerve fibers in the brain’s white matter are tracked by VFs whose global trajectories are streamlines to visualize nerve tracts.^21^ VFs have also been used to both assess the collective behavior of bacterial mobility and quantify this fluid-like motion using the Reynolds number, i.e., the ratio of inertial to viscous forces, as a metric.^22^ Thus, VFs analysis has been used to evaluate systems in diverse disciplines and has the potential to shed new insight in assessing the spatial organization of collagenous fibers.

In this paper, we propose a method to analyze SHG collagen images as pseudo-VFs and apply measures for assessment that are derived from fluid mechanics. In our approach, we first derive a 2D quadrilateral mesh that encapsulates pseudounit vectors in each cell based on preferred local orientations by FT-SHG analysis.^16^ The collection of these vectors is analogous to the velocity map of a fluid flow. Next, fluid-mechanics metrics such as enstrophy E, tortuosity τ, and energy U are used to characterize these VFs. E relates to the vorticity of a fluid flow and is used to assess circularly organized collagen fibers. τ correlates with the straightness of the fibers, and U captures the orientation changes of the VFs.

This paper is organized as follows. In Sec. 2, we present the methodology and samples preparation to evaluate fluid mechanic metrics in cow tendon, cortical porcine bone, and rat vaginal tissue. Section 3 reports and discusses the results in this study. Finally, Sec. 4 describes our conclusions and future work.

## 2 Materials and Methods

Bovine tendon was stored in phosphate-buffered saline (PBS) and sectioned with a cryostat (Thermo Scientific, NX50) at 200-μm intervals. The tissue sections were collected on glass microscope slides and imaged by SHG microscopy. Porcine femoral bones were cleaned from soft tissue and wrapped in PBS and frozen at −20°C. When ready for imaging, the samples were defrosted in PBS for 12 h, cut transversely into hollow cylinders from mid-diaphysis and polished from coarse to fine. Finally, rat vaginal tissue was obtained from virgin female Sprague-Dawley rats that were decapitated and frozen at −20°C. Before imaging, the samples were defrosted and hydrated with PBS at room temperature. Samples were sectioned along the axial length of the urethra. The dorsal side of the sample was laid flat and attached with cyanoacrylate adhesive to PBS-filled Petri dish. All samples were imaged using a commercial multiphoton microscope using an excitation wavelength at or near 780 nm; examples with experimental details can be found in our previous papers (see Refs. 6, 7, and 16). For all porcine and rat samples, at least three representative SHG images are selected for subsequent analysis. Bovine samples were obtained from a local abattoir. Porcine tissue preparation and imaging data were obtained according to a previously published report [see Ref. 6]. In this case, unpublished image data were used for this study. Rat vaginal samples were harvested with the approval of the Institutional Animal Care and Use Committee at Virginia Tech.

**Fig. 1:**
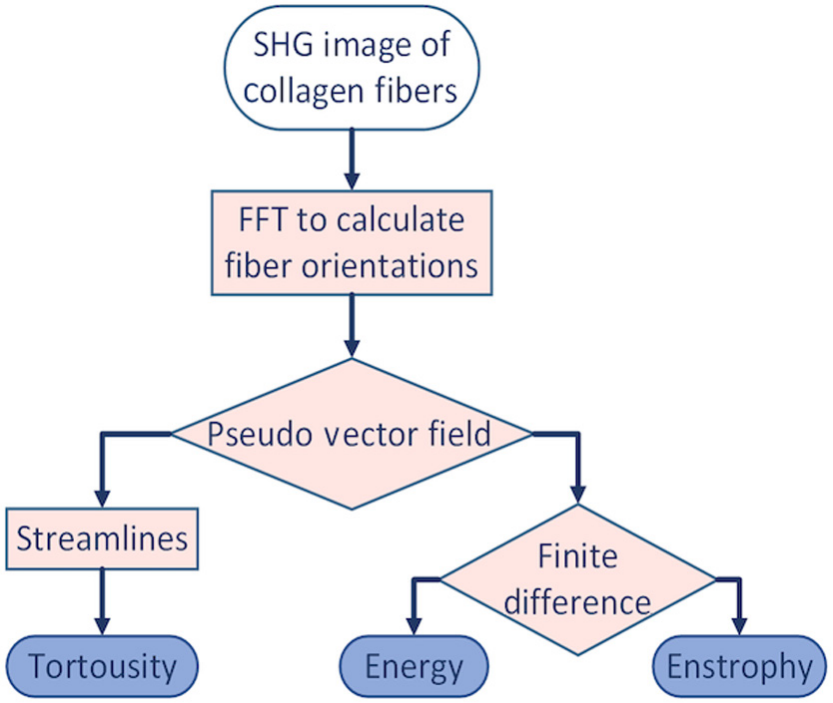
Tree diagram of the algorithm used to calculate τ, U, and E.

In characterizing the structure of collagen fibers, we consider the parameters τ, U, and E of the VFs. Acquired SHG images are converted to pseudo-VFs of unit length that represent preferred fiber orientation obtained by 2D Fourier transform analysis.^4^ Dark regions found from FT-SHG image analysis could be interpreted as objects around which a fluid flow. To calculate τ, which describes the presence of bends or twists in the fiber relative to being straight, VFs are first converted to streamlines. In fluid mechanics, streamlines are a set of lines tangent to the velocity vectors of a fluid flow. We point out that in our case the streamlines are not an exact facsimile of the original fibers because of the presence of dark regions (missing fibers) and isotropic regions (disorganized fibers) in the image. To account for this, we set each streamline to originate from every vector in a VF. Next, τ is calculated by quantifying the path P of streamlines tangent to the VF, over the distance D between its ends as described in Ref. 23. For the calculation of E and U, VFs are analyzed using 2D forward finite difference analysis.^24^ The definitions for τ, E, and U are

$$\tau =\frac{P}{D},$$ (1)

$$E=\int_{A}|\nabla \vec{\nu }{|}^{2}dA,$$ (2)

and

$$U=\int_{A}\rho dA,$$ (3)

where $\vec{\nu }$ represents the pseudovelocity field, ρ represents the density of the fluid which equals one for our pseudo-VF, ∇ is the curl operator, and A is the area comprising the VFs. Note that we employ a pseudo-VF to represent $\vec{\nu }$ as our static images do not capture a true velocity, but rather changes in fiber orientation (direction). Finally, we quantify the histogram of τ with the gamma γ distribution, a two-parameter-dependent frequency distribution.^25^ The amount of skew inversely depends on the shape parameter $\gamma_{a}$ and the proportion is dependent on the scale parameter $\gamma_{b}$. Calculations of fluid metrics E, U, and τ were performed in MATLAB. A summary of our algorithm is shown in Fig. 1.

**Fig. 2:**
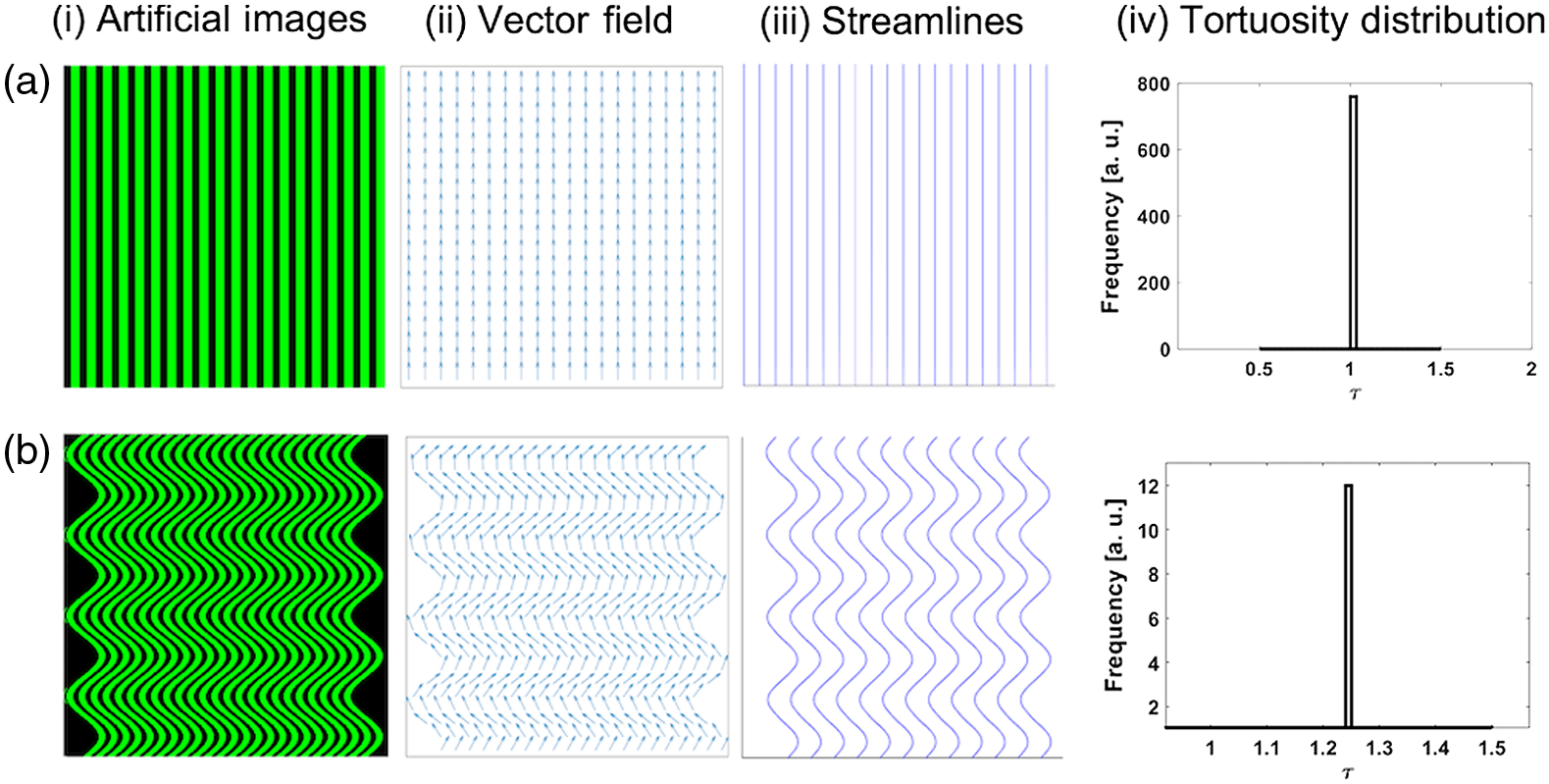
Representative synthetic images with (a) (i) uniformly aligned fibers and (b) (i) wavy fibers. (ii) The corresponding VF maps for each image, (iii) associated streamlines, and (iv) the histograms of tortuosity values.

To calibrate our method, we utilize digital phantoms generated in MATLAB. Figure 2(a, i) shows perfectly straight fibers, and the corresponding vector-field distribution and streamline data are shown in Figs. 2(a, ii) and 2(a, iii), respectively. In this case, as expected, we find τ to be 1, as shown in the histogram distribution in Fig. 2(a, iv). This model of straight fibers is an idealization, as natural collagen fibers, which may be uniformly organized generally do not exhibit rigid rod-like characteristics. We see in Fig. 2(b, i) our simulated representation of wavy fibers and the corresponding distributions in Figs. 2(b, ii) and 2(b, iii) as respective VFs and streamlines. Here, we find τ to be 1.25. U and E are calculated for these phantoms and the results are found to be 0 for both parameters for the straight fibers, and 191.9 and 106.0 for the wavy fibers, respectively.

## 3 Results and Discussion

**Fig. 3:**
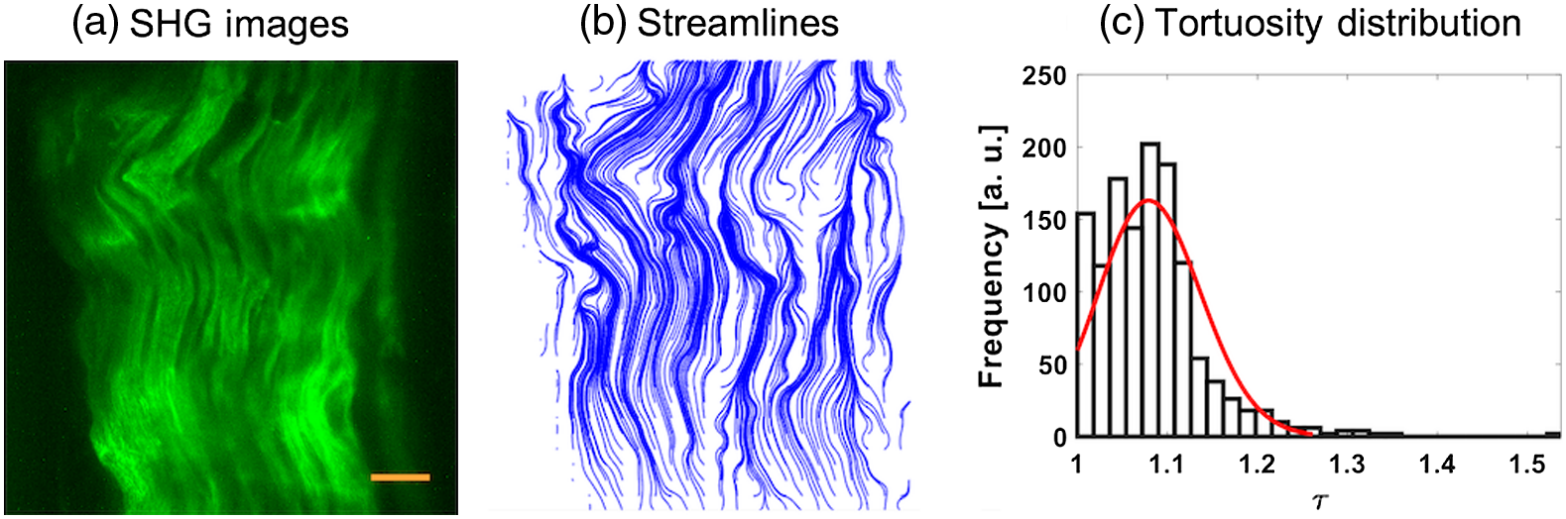
(a) Representative SHG image of bovine tendon. (b) The corresponding streamlines, (c) the distribution of the tortuosity values. The scale bars represent 25  μm.

FT-SHG images of bovine tendon, rat vaginal tissue, and porcine cortical bone are the subjects of this study. Bovine tendon has a relatively organized collagen fibrillar structure, and thus is a useful case study for understanding our algorithm to relatively straight fibers or in-plane wavy fibers. After applying our analysis on a bovine tendon SHG image [Fig. 3(a)], where a mixture of straight and wavy fibers is present, we observe streamlines generated from its VF [Fig. 3(b)] and that the distribution of τ ranged between 1 and 1.3, having values cluster around 1.1 [Fig. 3(c)]. As noted earlier, straight fibers result with τ tending toward 1, and generally the presence of straight and wavy fibers enlarges the range of τ. We find that this tissue results in values of U and E to be 284 and 302, respectively. While U and E are similar in mathematical definition, it is useful to interpret U as capturing the rapid changes in fiber directions such as what one observes for strongly crimped or wavy fibers, while E is more sensitive to circular, vortex-like organization of fibers.

**Fig. 4:**
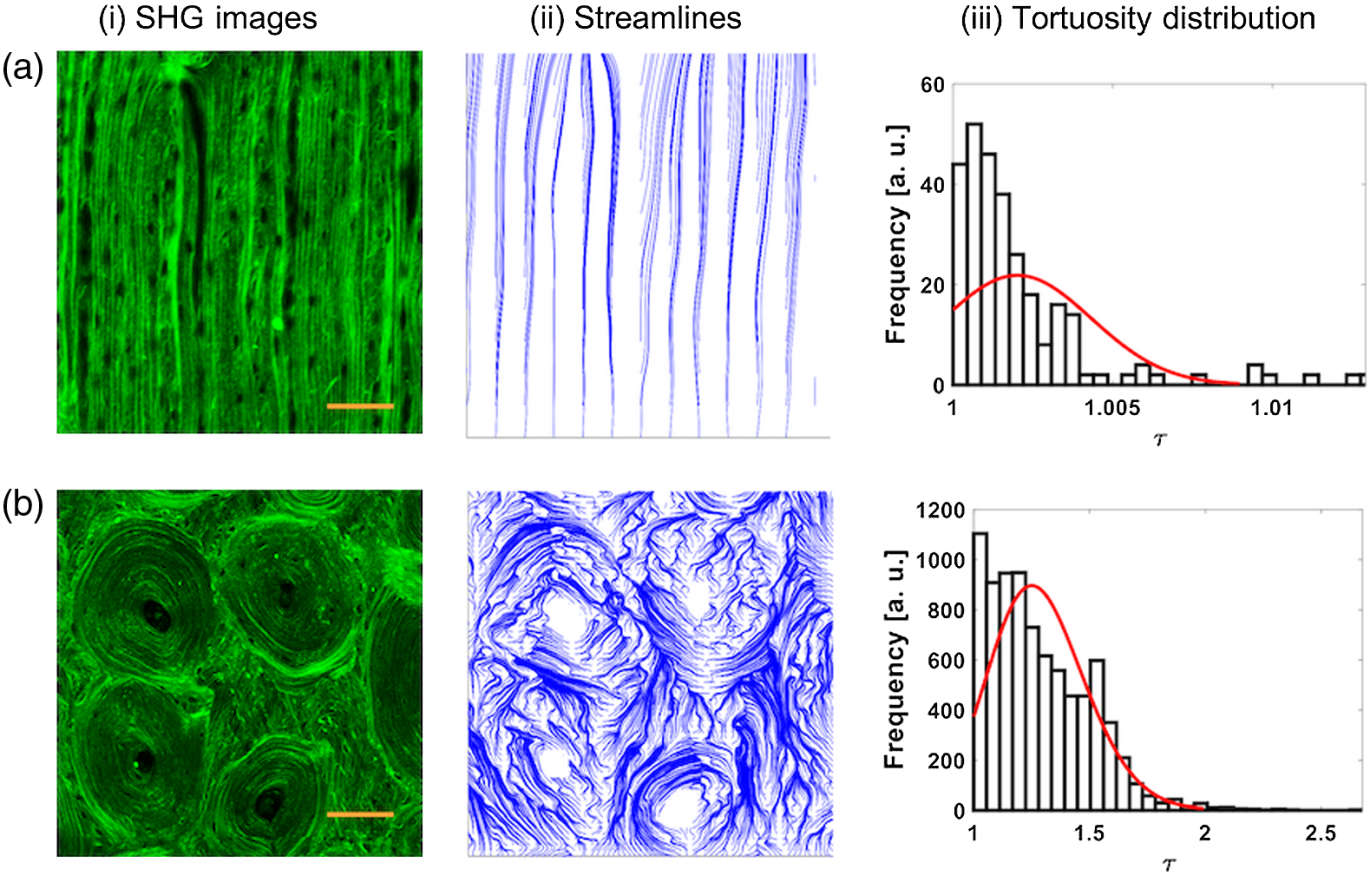
Representative SHG images of cortical bone with (a) (i) uniformly aligned fibers and (b) (i) Harversian canals. (ii) The corresponding streamline data for each image, (iii) the distribution of the tortuosity values. The scale bars represent 200  μm.

Furthermore, we apply a similar analysis to several porcine cortical bone SHG images, where fibers are uniformly aligned [Fig. 4(a, i)], as well as to images that display a vortex-like distribution when Harversian canals are present [Fig. 4(b, i)]. Streamlines and τ distributions are represented in columns (ii) and (iii). Streamlines shown in Fig. 4(a, ii) delineate the general shape of linearly organized cortical bone fibers. Hence, Fig. 4(a, iii) shows a small distribution of τ ranging from 1 to ∼1.007, with values clearly weighted toward 1, indicating the presence of mostly straight fibers. Our resulting calculations for U and E calculations are 5.70e−1±0.097 and 6.20e−1±0.067, respectively. In the case of samples with Harversian canals, τ values range from 1 to 2. The values of U and E calculated in this case are 2.58e3±262.7 and 2.64e3±270.8, respectively. The presence of Harvesian canals produces vortex-like behavior resulting in a clear magnitude difference for E and U.

**Fig. 5:**
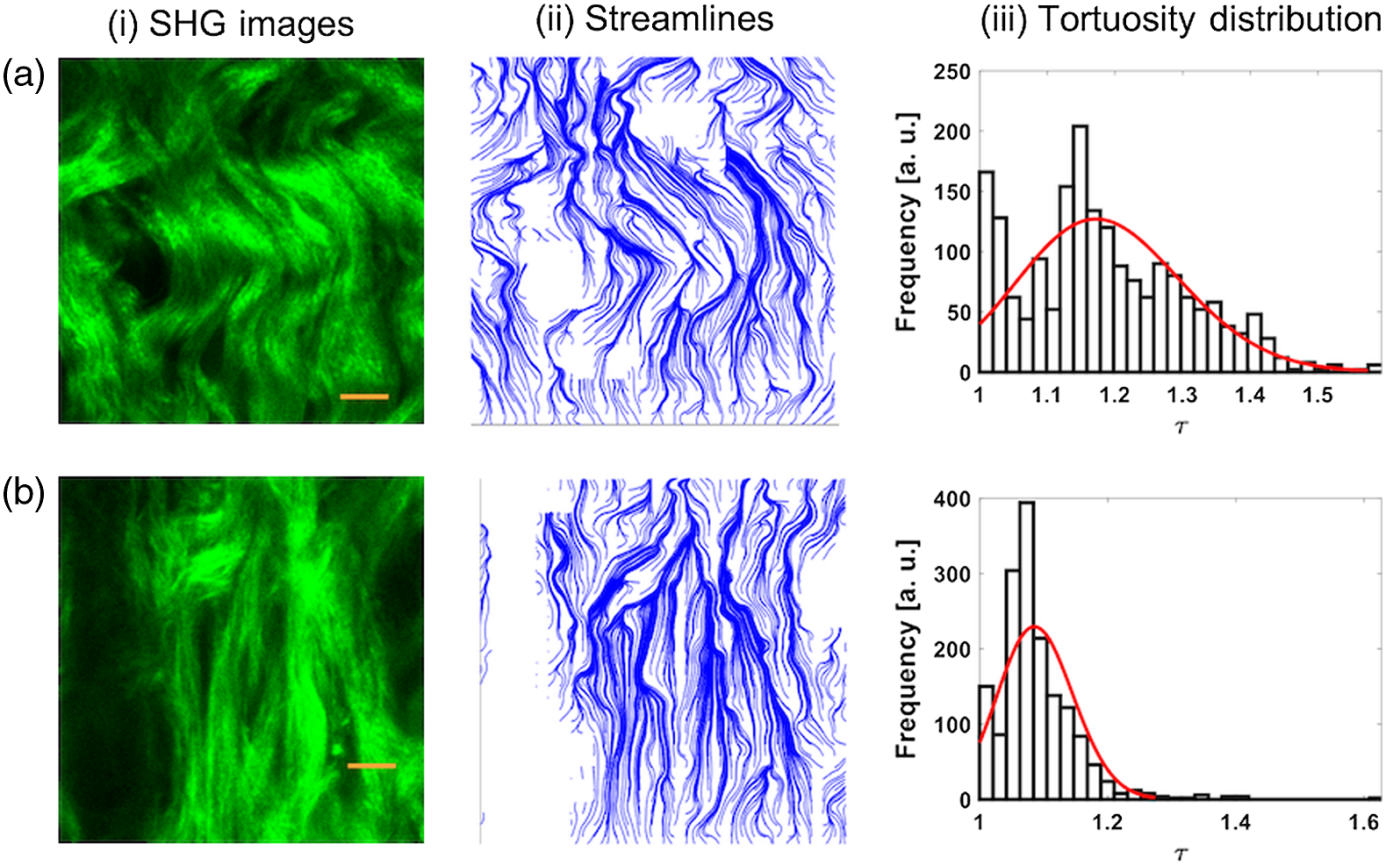
Representative SHG images of rat vaginal tissue close to the (a, i) cervix and (b, ii) introitus. (ii) The corresponding streamline data for each image and (iii) the tortuosity distribution values. The scale bars represent 10  μm.

Finally, representative images of rat vaginal tissue near the cervix [Fig. 5(a, i)] and introitus [Fig. 5(b, i)] regions are analyzed. We observe that fibers close to the cervix region tend to be wavier in comparison to fibers close to the introitus region where fibers are straighter. In addition, we show in Fig. 5(a, iii) that τ ranges from ∼1 to 1.5 and the values cluster around 1.17. E and U are determined to be 1.49e2±46.4 and 1.37e2±46.35, respectively. In contrast, SHG images nearby the introitus region have a range of τ values from 1 to 1.2, with the values tending to ∼1.1. In addition, we find the values of E and U to be 4.25e1±11.1 and 4.37e1±10.4, respectively. A summary of the values of E, U, τ, $\gamma_{a}$, and $\gamma_{b}$ for samples in this study is provided in Table 1.

**Table 1:** Values of U, E, and corresponding $\gamma_{a}\;$and $\gamma_{b}$ γ-distribution parameters from τ histogram distributions of phantom images, bovine tendon, cervical porcine bone, and rat vaginal tissue.

Sample	U	E	$\gamma_{a}$	$\gamma_{b}$
Straight phantom	0	0	Inf	0
Wavy phantom	1.92e2	1.06e2	Inf	0
Bone straight	5.70e−1±0.097	6.20e−1±0.067	6.34e5	1.58e-06
Near introitus vaginal tissue	4.25e1±11.1	4.37e1±10.4	3.40e2	3.20e-3
Near cervix vaginal tissue	1.49e2±46.4	1.37e2±46.35	9.86e1	1.20e-2
Bovine tendon	2.84e2	3.02e2	3.65e2	2.98e-3
Bone canals	2.58e3±262.7	2.64e3±270.8	3.86e1	3.33e-2

Samples chosen for this study have diverse architectures ranging from straight to vortex-like collagen-fiber organizations. We observe that E and U strongly correlate with collagen-fiber waviness. For instance, the values of E and U for regions close to the introitus of the rat vagina are less than those close to the cervix, thereby indicating that the latter is wavier. We observe a similar behavior when comparing the values of E and U for regions by the introitus to that of bovine tendon. Moreover, we confirm that the largest values of E and U in our study come from bone Harversian canals, where the vortex-like circulation of collagen fibers around these canals is observed. Furthermore, we can relate E and U values to the architecture of collagen fibers and categorize them into linear, wavy, and circular. Approximately straight fibers have values between 0 and 1, while circular fibers have values of E and U that begin around 80 and go out to about 3000. However, there are regions where distinguishing wavy and circular fibers from E and U alone become difficult, such as values of 80 and above, and thus τ would need to be included for differentiation. While, not surprisingly, the values for E and U appear to follow each other, we expect that they will begin to diverge when the analysis is applied to volumetric images of tissues, where the underlying collagen-fiber orientation alternates with depth, such as in the case of imaging cornea.^26^ The sensitivity of our approach to small changes in flow (directions of fibers) is dependent on the fiber resolution that we have when implementing our finite difference implementation, which is ultimately limited by both the optical resolution and computational cost.

From the γ-distribution analysis of τ, we see that $\gamma_{a}$ has larger values for uniformly aligned fibers in comparison to heterogeneously aligned fibers. In the analysis of biological fibers, each vector has a streamline associated with it that originates at the tail of the vector and follows the direction of the VF. Moreover, vectors are confined to the upper hemisphere (covering angles from 0 deg to 180 deg). As a result, the head of each vector will tend toward the top of the image, and the streamlines will thus follow this direction. Moreover, since the streamlines will have different starting points (based on the location of each vector), there is likely to be a higher density of streamlines at the top of the image with much less clustering at the bottom. This artifact will affect the histogram of τ in terms of both the peak and range of values. However, we find this approach to be useful for differentiating the specific range of collagen architectures presented in this paper. Finally, note that the metrics derived in our work are a direct analysis of VFs derived from local collagen fiber orientations and should not be confused with similar metrics used in texture analysis. Energy in texture analysis represents the homogeneity of the grayscale distribution and is derived from the method of gray level co-occurrence.^27^

## 4 Conclusions and Future Work

While current FT methods capture individual fiber orientation, the complexity of collagen architectures are not easily quantified by FT methods alone. An analytical approach based on fluid mechanics enables assessment of fiber orientation as part of a unified system of vectors akin to fluid flow. E and U help quantify this flow, and τ captures the waviness of streamlines. Thus, even though there is no new spatial information revealed through this method, collective assessment of fiber orientation provides new insight in understanding overall collagen-fiber organization in complex tissues. We applied fluid mechanics analysis to investigate collagen fiber organization of bovine tendon, porcine cortical bone, and rat vaginal tissue. We found that one can represent collagen fiber orientation as pseudovectors of unit length. Furthermore, we showed that E, U, and τ quantitatively capture and distinguish diverse collagen architectures. We applied this method to evaluate fibers that were straight, wavy, and followed a circular organization. We observed that straight fibers result in a distribution of τ with a small range, but with its values clustering around 1. It was shown that the range of τ scales with the waviness of collagen fibers. As U captures rapid changes in fiber orientation and E vortex-like organization, we have shown how these values bifurcate from low to high values depending on the fiber structure. We believe that modeling fiber organization as a VF and applying fluid mechanics metrics to quantify collagen architecture facilitates understanding of the role of the collagen microstructure for biological function. This could lead to a better understanding of the mechanical properties associated with disease, ECM remodeling and aging.

Finally, our approach could benefit from exploring multiphase fluid flow analysis to single out fibers for individual study if desired. In addition, we explored SHG imaging of a single plane, but we are currently exploring adapting to three-dimensional image stacks.
